# Efficacy of a Customized Three-Dimensional Printing Surgical Guide for Tibial Plateau Leveling Osteotomy: A Comparison With Conventional Tibial Plateau Leveling Osteotomy

**DOI:** 10.3389/fvets.2021.751908

**Published:** 2021-11-25

**Authors:** Jayon Kim, Jaeeun Ko, Jaehwan Kim, Anna Seo, Kidong Eom

**Affiliations:** ^1^Department of Veterinary Medical Imaging, College of Veterinary Medicine, Konkuk University, Seoul, South Korea; ^2^Research Institute, SEEANN Solution Co., Ltd., Incheon, South Korea

**Keywords:** 3D printing, computed tomography, dog, surgical guide, tibial plateau angle, tibial plateau leveling osteotomy

## Abstract

**Objective:** To prospectively evaluate the effect of a computed tomography (CT)-based three-dimensional (3D) printing surgical guide on surgical accuracy of tibial plateau leveling osteotomy (TPLO).

**Study Design:** Cadaveric study.

**Animals:** Canine cadaveric hindlimbs (*n* = 14).

**Methods:** TPLO was performed on cadaver hindlimbs disarticulated at the coxofemoral joint to compare and evaluate the conventional TPLO method (*n* = 7) with one that used customized 3D printing surgical guides (*n* = 7). The operation time and postoperative tibial plateau angle (TPA) of the osteotomy were evaluated. Moreover, the osteotomy inclination, torsion, and distance and the direction of eccentricity were assessed using CT reconstruction.

**Results:** Significant differences in the operation time (*p* < 0.001), postoperative TPA (*p* < 0.05), osteotomy inclination (*p* < 0.05), and osteotomy torsion (*p* < 0.05) were observed.

**Conclusion:** The use of TPLO surgical guide reduced the operation time and inaccurate osteotomy.

**Clinical Significance:** The surgical technique applied with a customized 3D printing surgical guide could be used to perform osteotomy and TPA adjustment more precisely than conventional TPLO.

## Introduction

Cranial cruciate ligament rupture is considered one of the common causes of hind limb lameness in dogs, and it results in stifle joint instability and progressive osteoarthritis (1–3). Many orthopedic surgical techniques have been developed for cranial cruciate ligament rupture to stabilize the stifle joint, alleviate pain, and minimize osteoarthritis (4, 5). Among these, tibial plateau leveling osteotomy (TPLO) is a common surgical technique that has been used for cranial cruciate ligament rupture and is being increasingly performed in small breed dogs as well as large breed dogs (6–9). The short- and long-term results of TPLO are more favorable than those of other techniques (6–8, 10–12). In TPLO, reducing the tibial plateau angle (TPA) to 5° is recommended to reduce or eliminate the cranial tibial thrust and minimize the strain on the caudal cruciate ligament (13–16). Following osteotomy at the proximal level of the tibia, the tibial plateau is rotated at an angle of 5° to apply a bone plate (15). With improvement in the understanding of TPLO, researchers have recommended osteotomy techniques, surgical instruments, and implants (17–22).

Performing the osteotomy parallel to the tibial plateau and perpendicular to the sagittal plane of the tibia in the craniocaudal direction is critical because inaccurate positioning and orientation of the osteotomy can induce limb malalignment and increase the risk of tibial tuberosity fractures (23, 24). Moreover, deviation of the center of the osteotomy from the planned center results in translation of the proximal tibial fragment and inaccurate alteration to TPA (17, 23, 25). Therefore, accurate osteotomy is critical to improve the success of TPLO and to reduce the risk of complications, and previous reports suggest that the use of guides is associated with more accurate osteotomy (26, 27).

Three-dimensional (3D) printing has been increasingly used in orthopedic surgery in both human and veterinary medicine (28–30). In veterinary medicine, a 3D-printed bone model was used in the orthopedic field for surgical planning and surgical simulation for clinical application (31–34). Furthermore, 3D reconstruction, using medical image processing software with computed tomography (CT) imaging data, is performed to develop patient-customized 3D printing surgical guides and implants. There have been reports on the use of custom cutting guides to improve the accuracy of osteotomy in surgeries, such as total knee replacement and tibial osteotomy in human and veterinary medicine (35–37). Moreover, a 3D printed patient specific drill guide has been applied for the placement of transpedicular screws in canine cervical vertebrae (38). The use of the aforementioned guides improves the accuracy of the positioning of implants and surgical results and can influence surgical outcomes. These patient-specific surgical guide systems designed using 3D data derived from CT are commercially available to veterinary surgeons today to help achieve more accurate surgical outcomes.

The purpose of the present study was to create a customized TPLO surgical guide capable of effective and accurate osteotomy using 3D printing technology and to describe the surgical technique. Additionally, the efficacy of the TPLO surgical guide compared to that of the conventional TPLO method was prospectively investigated using a CT-based evaluation method.

## Materials and Methods

### Study Population

Fourteen cadaver hindlimbs without the cranial cruciate ligament were obtained from seven beagle dogs euthanatized for reasons unrelated to this study. The cadaver hindlimbs were disarticulated at the coxofemoral joint. This study was approved by the Institutional Animal Care and Use Committee of Konkuk University (Approval no. KU21039).

### Preparation of the Surgical Guide

CT images of the hindlimbs were obtained using a 4-channel helical CT scanner (LightSpeed, GE Healthcare, Milwaukee, WI). The scanning parameters were as follows: 120 kVp, 200 mAs, 1.25 mm in slice thickness, and bone algorithm. CT images were taken with 90° of the stifle joint and tarsal joint flexion. All images were evaluated using digital imaging and communication in medicine (DICOM) files, and loaded onto post-processing software (Radiant, Medixant, Poznan, Poland). The TPA was measured during preoperative planning. The intercondylar tubercle, which is the center of osteotomy, was identified and the difference in the angle at which the postoperative TPA becomes 5° was calculated.

**Figure 1:**
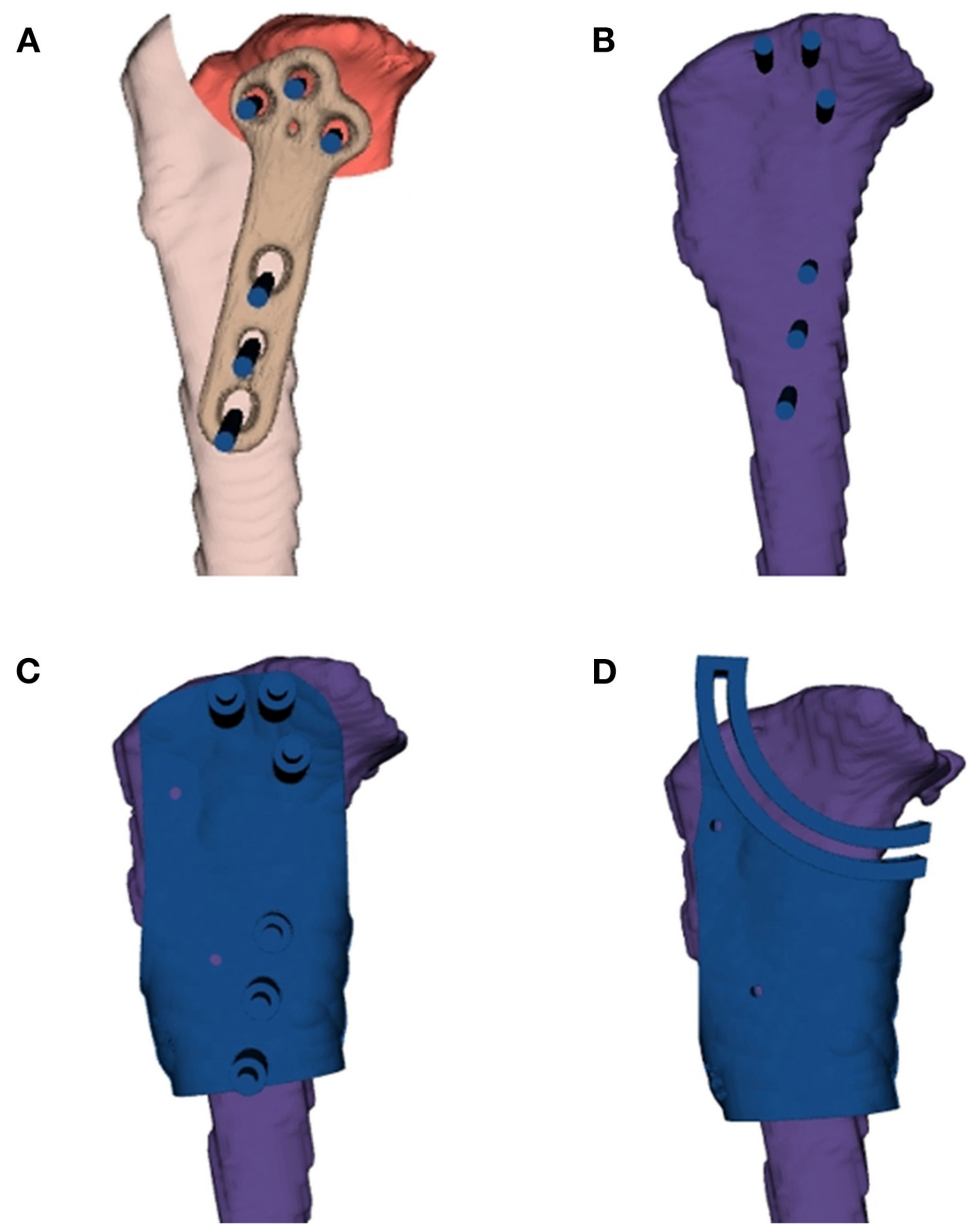
Medial aspect of the virtual tibia model for the customized three-dimensional printing surgical guide design. **(A)** The proximal tibial plateau segment is virtually rotated. **(B)** A TPLO plate is placed and a virtual trajectory is created according to the screw insertion direction. **(C)** The rotated proximal tibial plateau segment is placed back, and a drill guide template is created corresponding to the screw trajectory. **(D)** A saw guide template is created with the diameter based on the preoperative planning. TPLO, tibial plateau leveling osteotomy.

**Figure 2:**
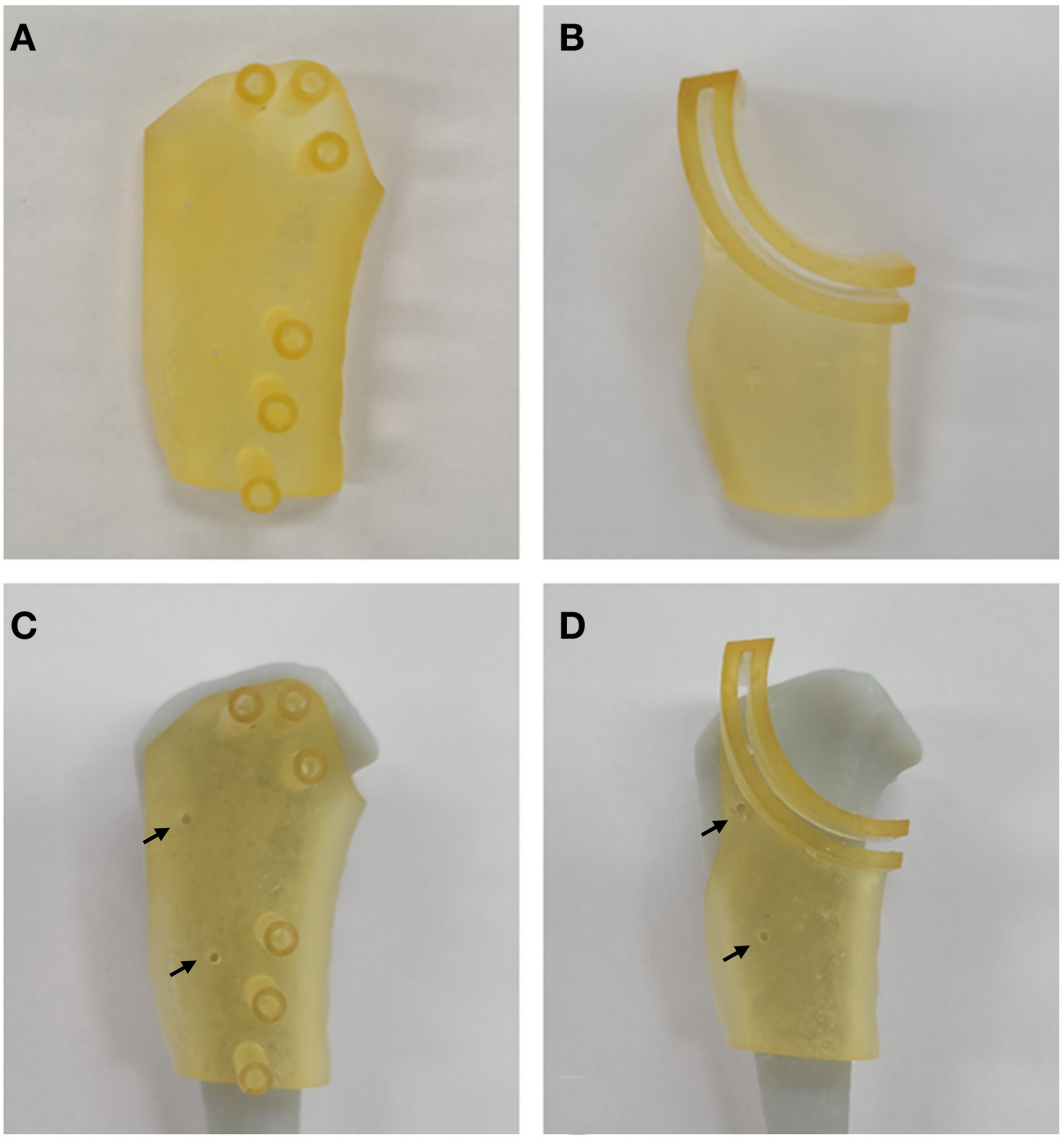
Customized three-dimensional printing surgical guides. TPLO drill guide **(A,C)** and TPLO saw guide **(B,D)**. Arrows represent the two holes for temporary fixation. TPLO, tibial plateau leveling osteotomy.

DICOM images from the CT were exported to a medical image processing software (Reconeasy, SEEANN solution, Seoul, Korea) for 3D reconstruction of the tibia for surgical simulation. The TPLO surgical guide consisted of a TPLO drill guide and saw guide. The design procedure was as follows: the proximal tibial plateau segment was virtually rotated by an angle previously calculated using the diameter of the saw blade used in surgery. A TPLO plate of the same size that was to be used for surgery was placed, and virtual trajectory was created with the corrected position and angle according to the screw insertion direction. The diameter of the virtual trajectory was similar to that of the drill bit. The rotated proximal tibial plateau segment was placed back to its original position to create a drill guide template, corresponding to the screw trajectory. A saw guide template was created according to the osteotomy line from the center of the osteotomy, based on the diameter of the saw blade determined during preoperative planning (Figure 1). Each drill and saw guide template was designed to fit the anatomical structure. Therefore, the guide templates could be placed at positions similar to those during simulation. Moreover, the templates had some holding force because they were partially settled on the surrounding anatomical structures. Two additional holes to temporarily fix the drill guide and the saw guide during the surgery were made at a similar location for additional fixation (Figure 2).

The TPLO surgical guides were printed by a 3D printer (**Figure 4** standalone, 3D system Inc., Rock Hill, SC) with biocompatible and autoclavable material for medical use (**Figure 4** MED-AMB 10, 3D systems Inc., Rock Hill, SC).

### Surgical Technique

TPLO was performed on the cadaveric tibias (*n* = 14). While group A (*n* = 7) underwent a TPLO with the typical surgical method using a Slocum jig, group B (*n* = 7) underwent TPLO using a customized 3D printing surgical guide.

**Figure 3:**
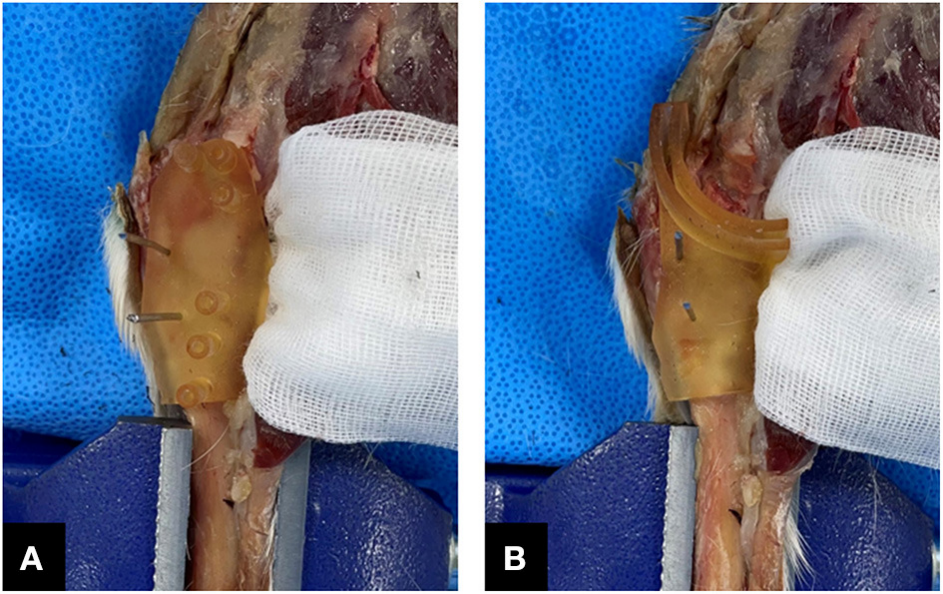
Application of the customized three-dimensional printing surgical guides intraoperatively. TPLO drill guide **(A)** and saw guide **(B)** are temporarily fixed by two Kirschner wires. TPLO, tibial plateau leveling osteotomy.

All TPLO procedures were performed by one surgeon. The surgical technique using the TPLO surgical guide is as follows. A drill guide was applied with two Kirschner wires. After drilling along the hole in the drill guide, temporary pins and the drill guide were removed. The saw guide was applied with two Kirchner wires at a similar position where the drill guide was temporarily fixed. Following osteotomy along the saw guide, the temporary pins and guides were removed (Figure 3). The osteotomy was stabilized with a TPLO plate (Biortho, Jiangsu, China) and screws along the pre-drilled hole. Radiography (Titan 2000V, Comed Medical system, Seoul, Korea) and CT imaging (120 kVp, 150 mAs, 0.625 mm in slice thickness, and bone algorithm) were performed postoperatively.

### Assessment of the Operation Time

The time from applying the jig to the time of removing the temporary fixation pin after plate and screw application was measured in group A. In contrast, the time from applying the drill guide to the time of applying the plate and screw was measured in group B. Data were analyzed for significant differences in the operation time between the two methods.

### Assessment of the Tibial Plateau Angle

All imaging measurements were evaluated by one radiologist. Postoperative TPA was randomly measured three times. On the mediolateral tibial radiographs, the angle between the line perpendicular to the tibial long axis and the tibial plateau axis was measured. CT images were reconstructed via the maximum intensity projection (MIP) technique by identifying the anatomical structure using a DICOM viewer and multiplanar reformation (MPR), positioning the stifle joints close to true lateral views. Moreover, the TPA was measured in a way similar to that with radiography. Data were analyzed for significant differences in the postoperative TPA between the two methods.

### Assessment of Osteotomy Accuracy for Inclination

**Figure 4:**
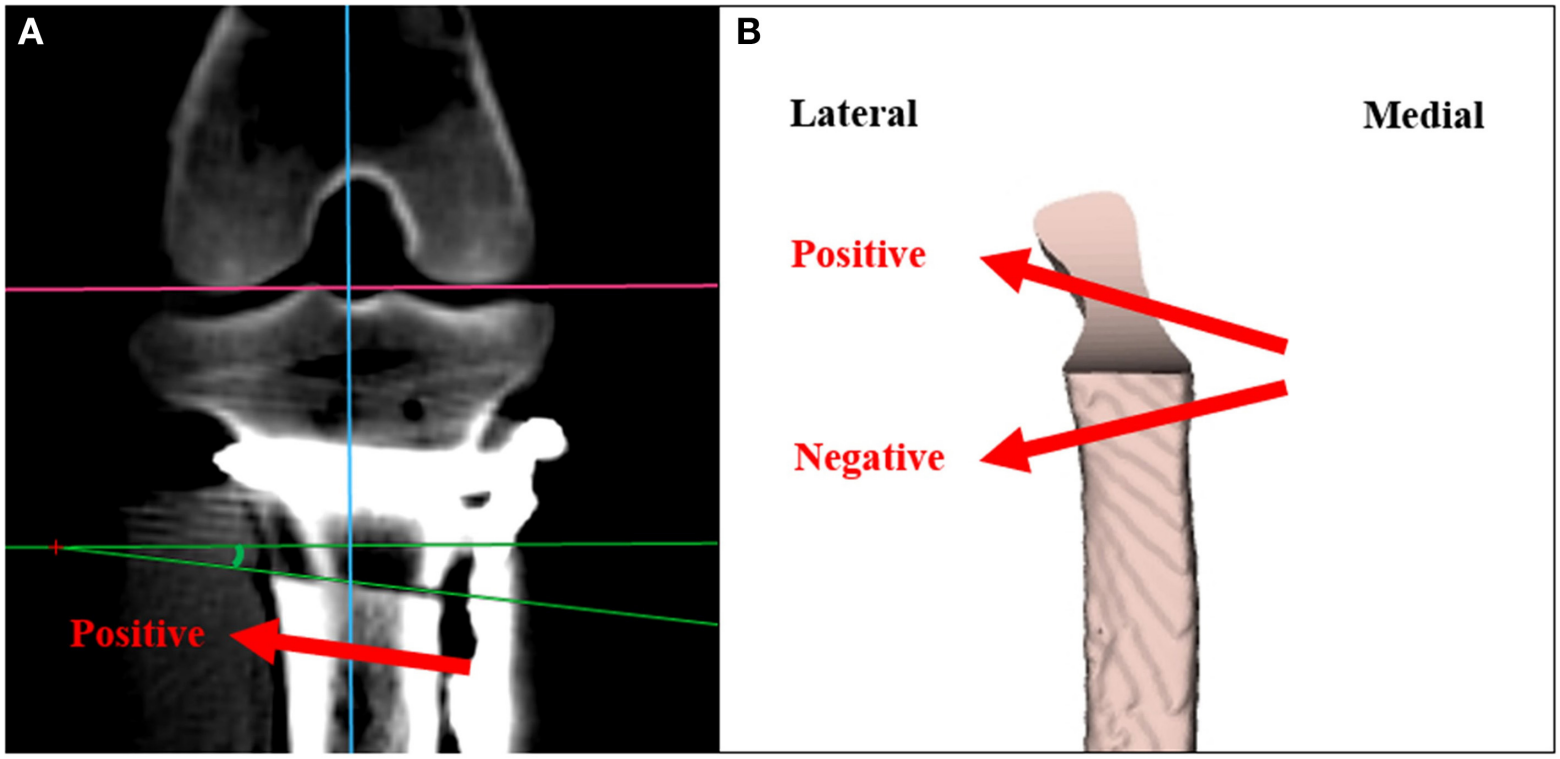
Measuring the angulation of osteotomy for inclination. **(A)** The angle of intersection of a line parallel to the osteotomy and a line with accurate orientation of the osteotomy is defined as the osteotomy inclination angle. **(B)** Positive angulation is defined as the direction of the osteotomy proximally from the medial cortex to the lateral cortex. Negative angulation is defined as the direction of the osteotomy distally from the medial cortex to the lateral cortex.

Postoperative CT images were measured on the dorsal plane with true lateral view of the positioning of the stifle joint using a DICOM viewer and MPR. Osteotomy inclination angle was calculated from the intersect of a line parallel to the osteotomy and a line in the accurate orientation of the osteotomy (Figure 4). The direction of the osteotomy proximally from the medial cortex to the lateral cortex was defined as a positive angulation. The direction of the osteotomy distally from the medial cortex to the lateral cortex was defined as a negative angulation (26). Data were analyzed for significant differences in the osteotomy inclination angle between the two methods.

### Assessment of Osteotomy Accuracy for Torsion

**Figure 5:**
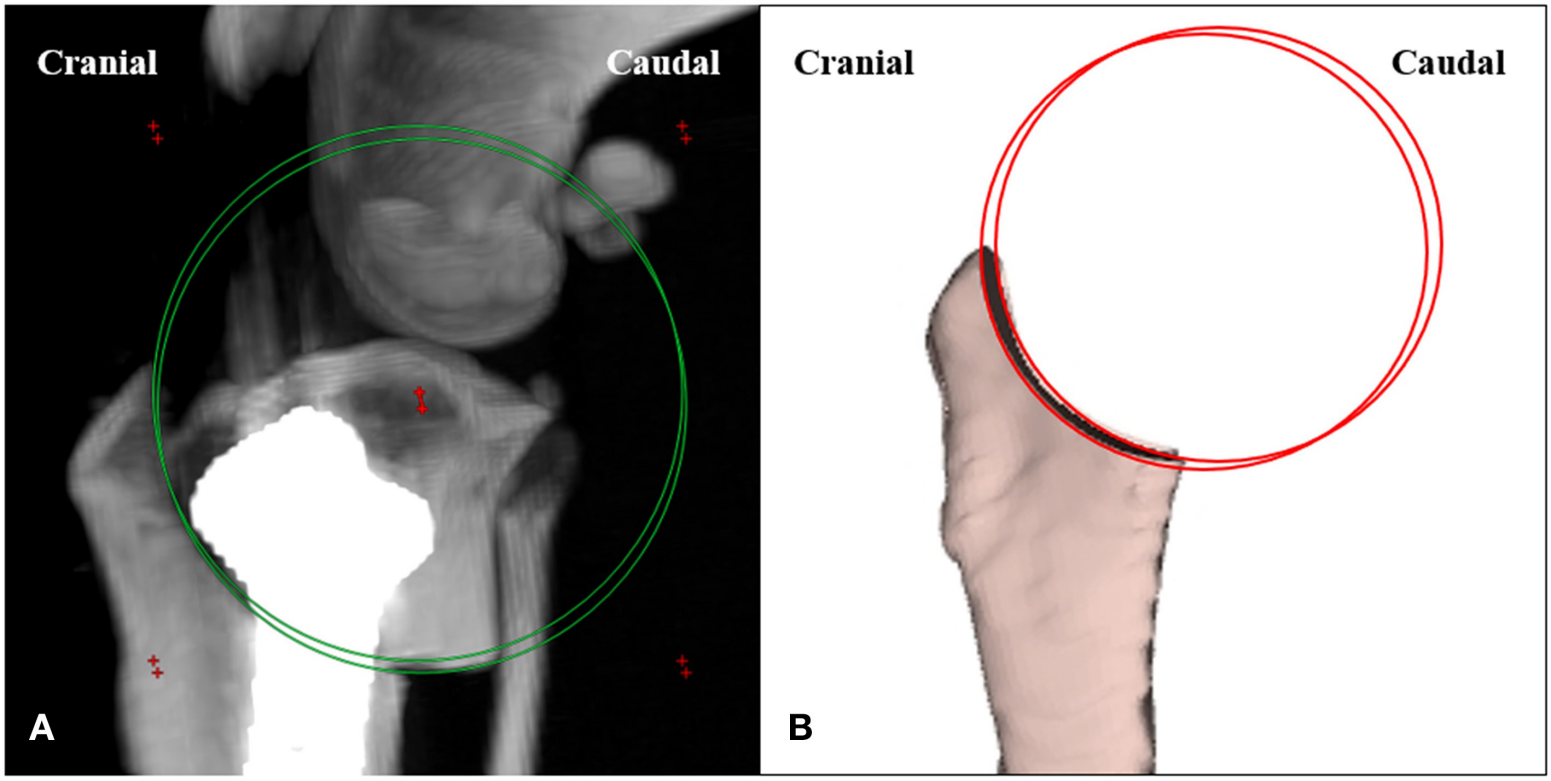
Measuring the angulation of osteotomy for torsion. **(A)** The distance between the center of the two circles of the corresponding saw blade radius superimposed over the osteotomy from medial and lateral aspects is defined as the osteotomy torsion. **(B)** The osteotomy lines are identified on the medial and lateral aspects.

Postoperative CT images were reconstructed with the MIP technique, positioned close to the true lateral view, and measured on the transverse plane. Osteotomy torsion was measured from the distance between the center of the two circles of the corresponding saw blade radius, superimposed over the osteotomy from the medial and lateral aspects (Figure 5). Data were analyzed for significant differences in the osteotomy torsion between the two methods.

### Assessment of Eccentricity of the Osteotomy

**Figure 6:**
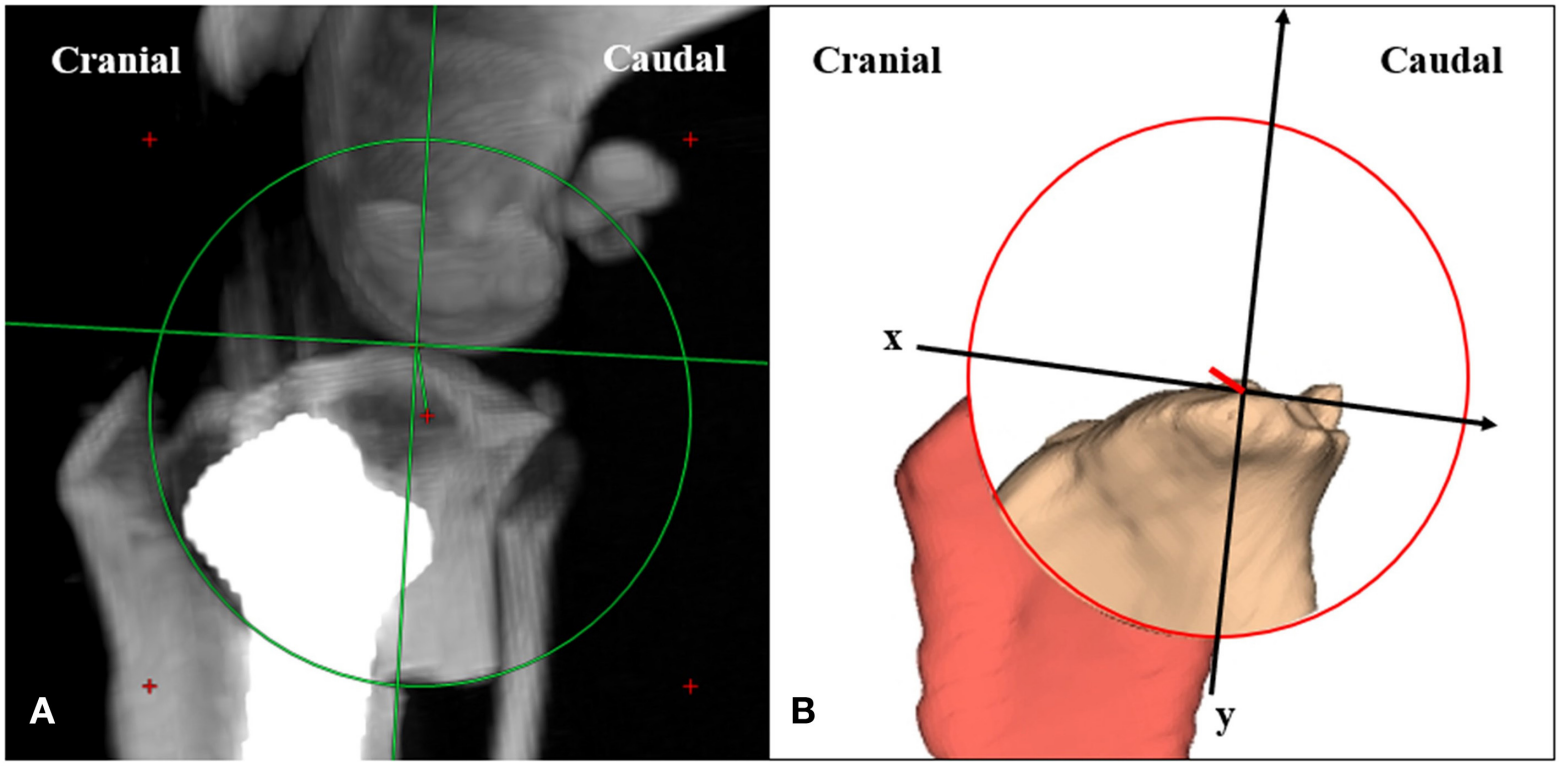
Measuring the distance and the direction of eccentricity. **(A)** The distance between the intended center of the osteotomy at the tibial long axis and the center of the actual osteotomy is defined as the distance of eccentricity. **(B)** The tibial long axis is defined as y-axis. The x-axis is defined perpendicular to the tibial long axis.

Postoperative CT images were reconstructed with the MIP technique, positioned close to the true lateral view, and measured on the transverse plane. Distance of eccentricity was measured from the distance between the intended center of the osteotomy at the tibial long axis and the center of the actual osteotomy (Figure 6). While the y-axis represented the tibial long axis, the x-axis was perpendicular to the tibial long axis. Additionally, the eccentric direction of the distance to the center of actual osteotomy was evaluated (27, 39). Data were analyzed for significant differences in the distance of eccentricity between the two methods.

### Statistical Analyses

Statistical analyses were performed using commercially available software programs (R version 4.0.4., The R Foundation for Statistical Computing, Vienna, Austria). *T*-test was used to compare the operation time, osteotomy inclination angle, osteotomy torsion, and the distance of eccentricity between the two methods. Normality and equal variance were assumed for the *t*-test, considering the insignificant results from the Shapiro-Wilk normality test and *F*-test. Comparison of the difference in TPA after surgery between the two surgical methods, according to the TPA measurement method, was examined by linear mixed models. For all comparisons, the statistical significance level was set at *p* < 0.05.

## Results

The operation time was significantly greater in the conventional TPLO group (30.21 ± 1.56 min) than in the TPLO surgical guide group (19.65 ± 1.29; *p* < 0.001) (Table 1).

**Table 1:** Comparison of the operation time, osteotomy inclination angle, osteotomy torsion, and distance of eccentricity according to the surgical method.

	**TPLO surgical method**		***P*-value**
	**Group A**	**Group B**	
Operation time (minute)	30.21 (1.56)	19.65 (1.29)	<0.001^*^
Inclination (degree)	5.51 (1.79)	2.76 (1.94)	0.017^*^
Torsion (mm)	1.08 (0.17)	0.58 (0.35)	0.005^*^
Eccentricity (mm)	3.04 (1.85)	2.54 (0.82)	0.525

*All values are presented as mean (standard deviation)*.

*TPLO, tibial plateau leveling osteotomy*.

* *p <0.05 is considered statistically significant*.

**Figure 7:**
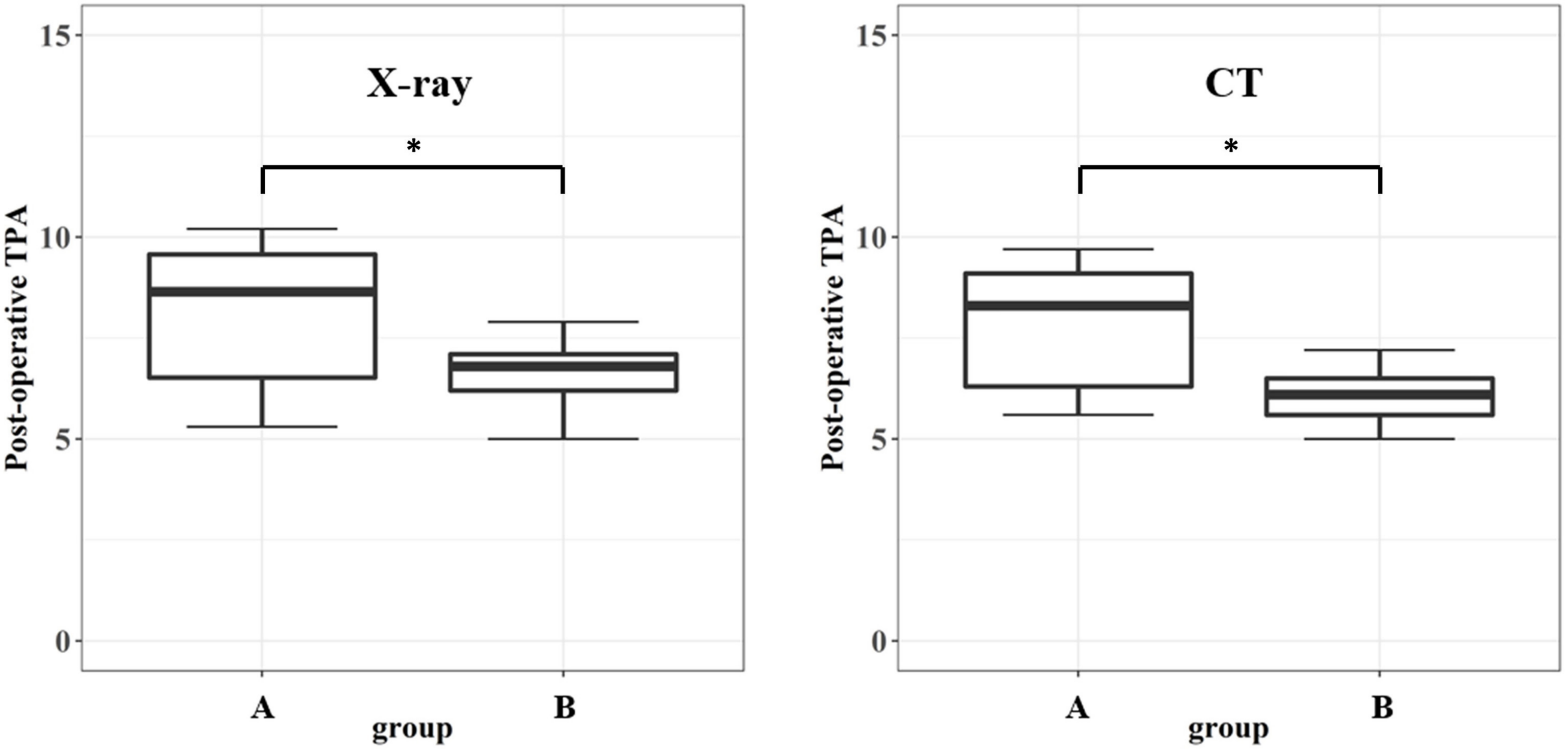
Postoperative tibial plateau angle for the two groups. The conventional TPLO method group has significantly greater postoperative tibial plateau angle than the surgical guide group in both modalities. TPLO, tibial plateau leveling osteotomy. *p < 0.05 is considered statistically significant.

The conventional TPLO group had a significantly greater postoperative TPA than the TPLO surgical guide method group on measurement with both radiography and CT (*p* < 0.05) (Table 2). Moreover, both radiography and CT revealed that the mean deviation of the postoperative TPA from the intended 5° was significantly greater in the conventional TPLO group than in the TPLO surgical guide group with (*p* < 0.05) (Table 2; Figure 7).

**Table 2:** Comparison of the postoperative tibial plateau angle and deviation according to the surgical method.

	**Method**	**Group**		***P*-value**
		**Group A**	**Group B**	
Postoperation TPA	X-ray	8.21 (1.65)	6.58 (0.78)	0.040^*^
	CT	7.89 (1.48)	6.09 (0.62)	0.015^*^
Postoperation TPA deviation from 5**°**	X-ray	3.21 (1.65)	1.58 (0.78)	0.040^*^
	CT	2.89 (1.48)	1.09 (0.62)	0.015^*^

*All values are presented as mean (standard deviation)*.

*TPA, tibial plateau angle; CT, computed tomography*.

* *p <0.05 is considered statistically significant*.

**Figure 8:**
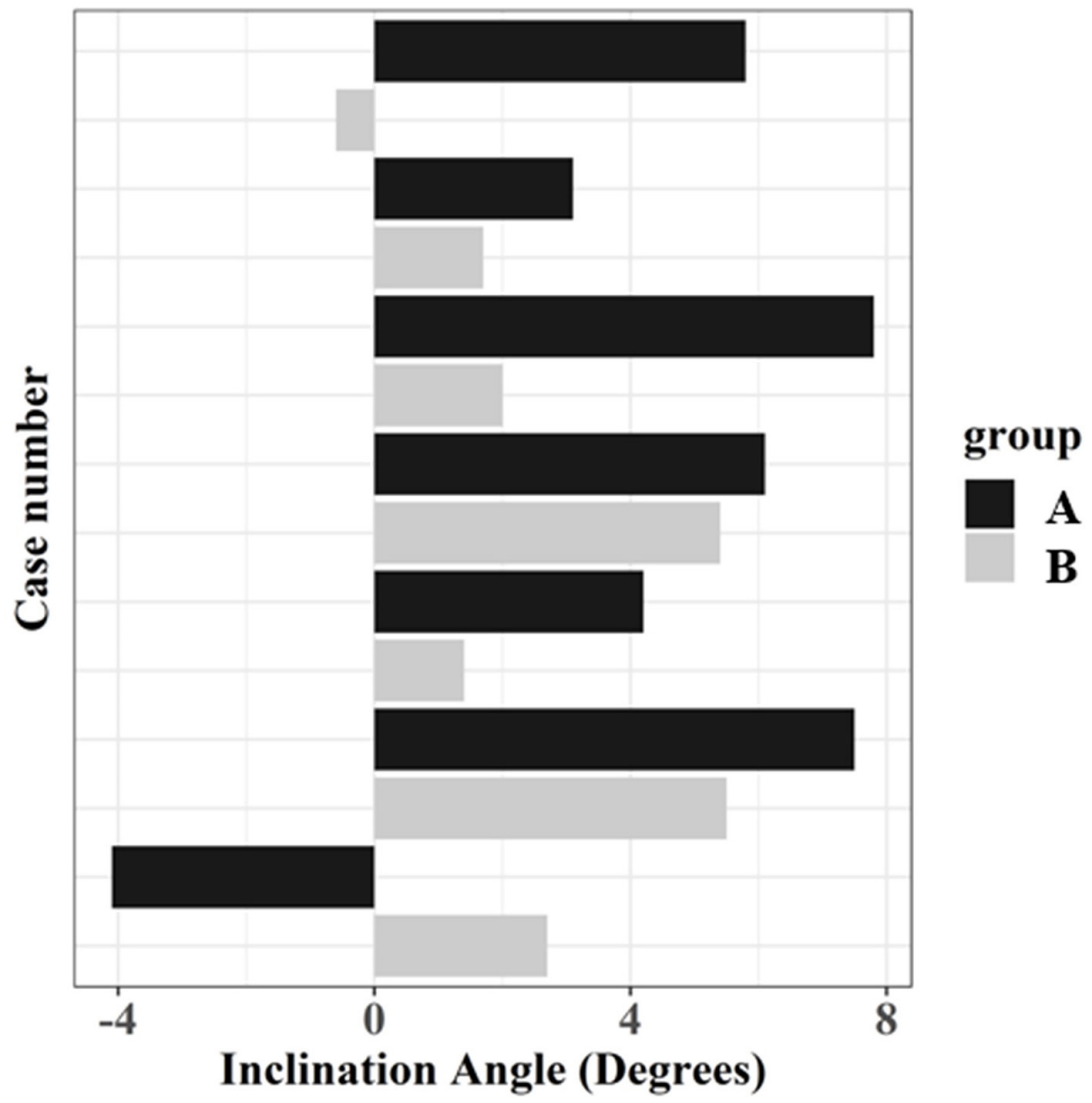
Osteotomy inclination angle for the two groups. The direction of osteotomy proximally from the medial cortex to the lateral cortex is defined as a positive angle. In contrast, the direction of the osteotomy distally from the medial cortex to the lateral cortex is defined as a negative angle.

The inclination angle revealed a significant difference between the groups (*p* < 0.05) (Table 1). Osteotomy performed with the TPLO surgical guide was more perpendicular to the tibial long axis. Figure 8 depicts the distribution of osteotomy inclination.

**Figure 9:**
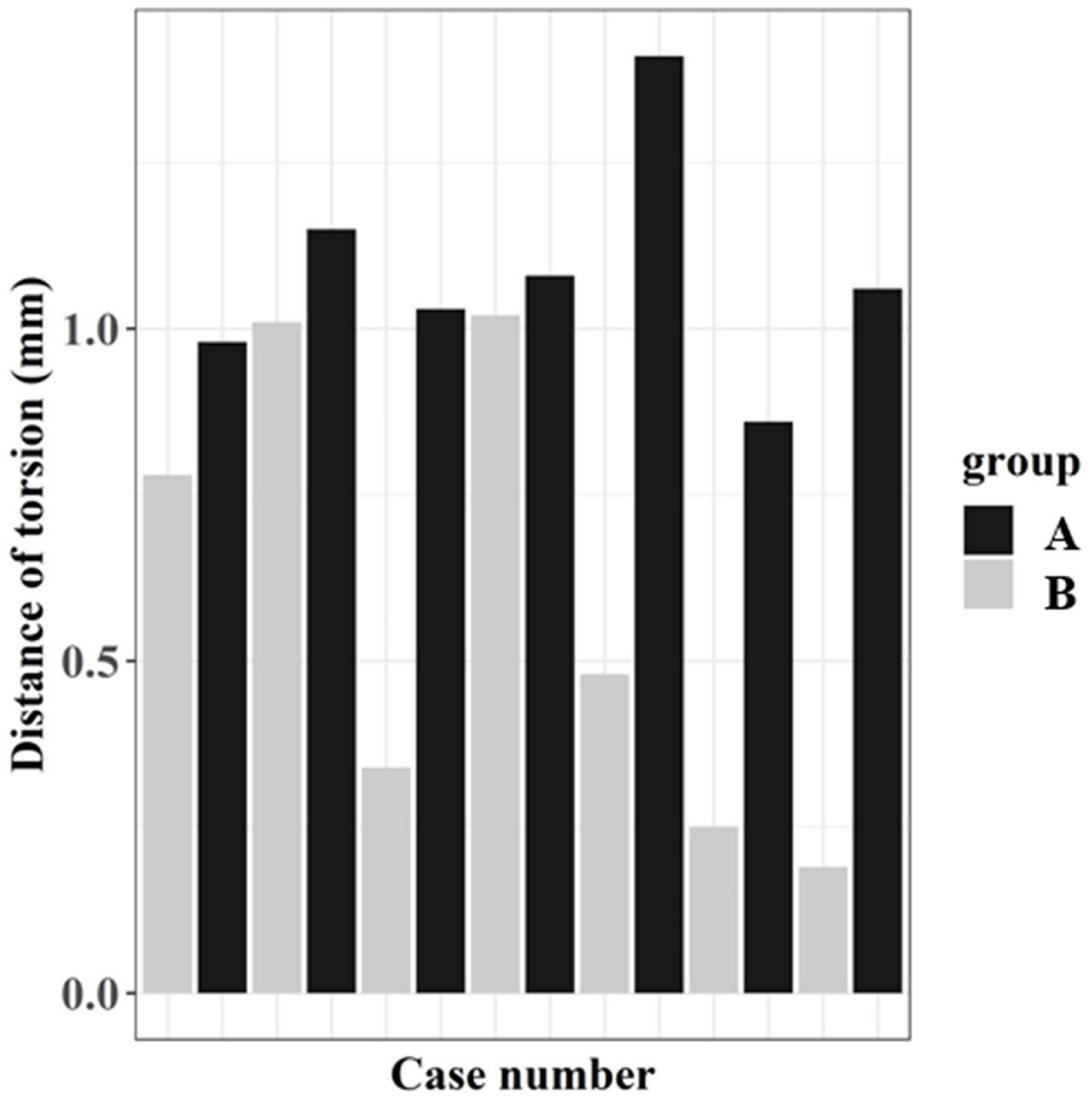
Osteotomy torsion distance measurement for the two groups. Osteotomy torsion is defined as the distance of the center of the two circles superimposed over the osteotomy from the medial and lateral aspects.

The osteotomy torsion distance revealed a significant difference between the groups (*p* < 0.05) (Table 1). Osteotomy performed with the TPLO surgical guide was more perpendicular to the tibial long axis. Figure 9 depicts the distribution of osteotomy torsion.

**Figure 10:**
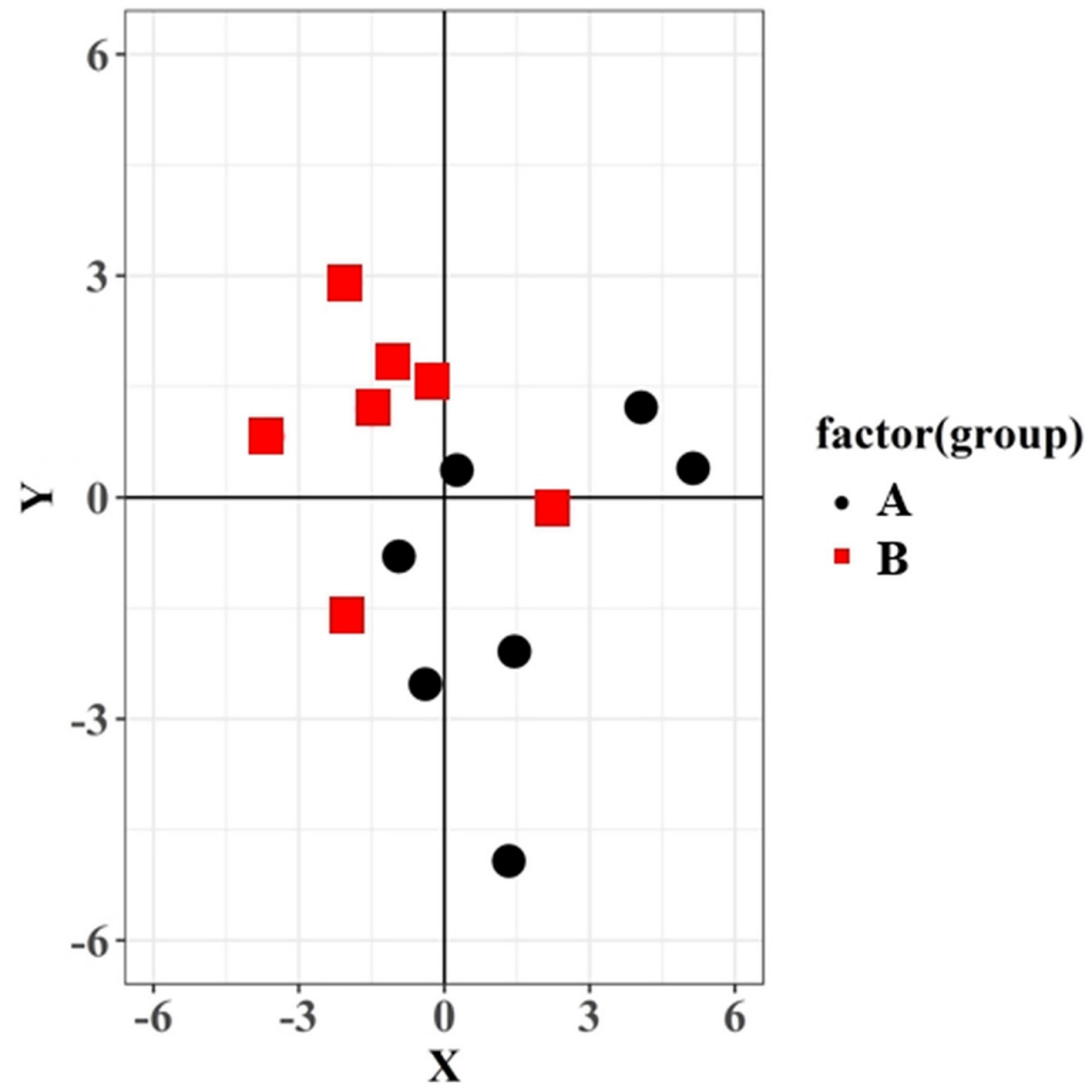
Distance and direction of eccentricity for the two groups. The y-axis represents the tibial long axis, and the x-axis represents perpendicular to the tibial long axis. The intersection of the x-axis and y-axis is the intended center of the osteotomy, and each data point represents the center of actual osteotomy.

The mean distance of eccentricity was not significantly different between the groups (*p* = 0.525) (Table 1). The actual center of osteotomy was located distally in 4/7 (57%) cases in the conventional TPLO group, but proximally in 5/7 (71%) cases in the TPLO surgical guide method group. Moreover, the actual center of osteotomy was located caudally in 5/7 (71%) cases in the conventional TPLO group, and cranially in 6/7 (86%) cases in the TPLO surgical guide group (Figure 10).

Regarding the subjective evaluation of the surgeon, tissue handling was less when the TPLO surgical guide was used. The surgical guide was designed to use the tissue handling space used to place and apply the implant in the conventional TPLO method. Therefore, more tissue handling for positioning the TPLO surgical guide was not required, as opposed to the conventional TPLO method that involves more tissue handling in the process of properly positioning the plate and drilling.

## Discussion

Accurate osteotomy depends on accurately implementing the preoperative planning during the operation (40) and performing the osteotomy precisely at the intended location. Studies comparing the conventional jig system and saw guides in veterinary medicine have been reported to accurately perform osteotomy (26, 27). These previous reports have associated the use of these guides with more accurate osteotomy. The present study identified several potential advantages of the TPLO surgical guide compared to the conventional TPLO method using Slocum jig. The surgical guide reduced the operation time as compared to that of the conventional TPLO method. This can be attributed to the possibility of reducing the time to mark the distance of the landmark during surgery and the time to drill by arranging the plate while using the drill guide.

The postoperative TPA was influenced by various variables, such as the preoperative TPA measurement, degree of bone segment rotation, distance and direction of eccentricity, and movement during plate application (27, 41). The TPLO surgical guide group demonstrated significantly less deviation in the postoperative TPA from the intended 5°, compared to the conventional TPLO group. The influence of factors affecting the postoperative TPA was possibly less while using the surgical guide than the conventional technique. This necessitates further evaluation of the TPLO surgical guide variables for better postoperative TPA outcomes.

The advantage of the TPLO surgical guide method is that it allows a more orthogonal approach to the long axis of the tibia and less angulation, such as inclination and torsion. The greater the degree of angulation, the greater the risk of intra-articular screw placement. Moreover, it may affect plate placement and increase the risk of tibial tuberosity fracture because of reduced bone volume in the tibial tuberosity (24, 26). Additionally, a non-orthogonal osteotomy can result in valgus and varus malalignment of the tibia, besides causing an over-rotation or under-rotation of the tibial plateau with a caudal or cranial long axis shift that may influence postoperative TPA (17, 42–44). Therefore, surgeons should strive to perform an anatomically accurate osteotomy. The TPLO surgical guide facilitates more accurate osteotomy than the conventional method.

The difference in the distance of eccentricity from the center of the intended osteotomy to the center of the actual osteotomy did not reach significance. However, the mean distance of eccentricity in the present study was similar to that previously reported (39, 45). The conventional TPLO method displayed a tendency for the direction of eccentricity to the caudal and distal deviation, consistent with the findings of a previous study (27). The reason for this is not clear, but the surgeon presumably tended to select the osteotomy center caudally and distally because of the presence of the proximal jig pin or to properly ensure the bone segment to appropriate stabilization with a bone plate (27). Conversely, when a TPLO surgical guide is used, the direction of eccentricity tended to be positioned in the opposite direction to the conventional TPLO method. The cranial and proximal deviation were considered displacements in the cranial and proximal directions, in which the saw guide could move because of the slight shaking while using a saw guide. The failure of the actual osteotomy center to coincide with the intended osteotomy center results in a disagreement regarding the effect of the tibial long axis shift on the postoperative TPA. Moreover, depending on the direction of eccentricity, the tibial tuberosity width can be reduced, thus potentially increasing the risk of tibial tuberosity fractures (46, 47). Therefore, an accurate osteotomy position is important because the distance and direction of eccentricity can affect postoperative complications. Considering the small number of cases in each group, it is necessary to further investigate if there is a significant difference in the distance and direction of eccentricity in a larger study.

The surgical guides provided additional advantages of simplifying the surgical technique and reducing tissue handling, as in human medicine (48). However, the present study was not applied to clinical cases, underscoring the need to further investigate whether these findings can be replicated in clinical cases. The simplification of the surgical technique reduced the operation time. Moreover, the use of the surgical guide ensured a stable position of the implant, which in turn reduced surgical complications. Therefore, even beginners in TPLO are expected to reduce complications that may be caused by their inexperience.

The use of TPLO surgical guides had several disadvantages. The procedure requires anesthesia for CT scans. However, the CT scans enabled more accurate preoperative planning, and the anesthesia time during surgery could be reduced. Another disadvantage was the need for essential experience with the medical image processing software and 3D printing hardware. It requires time to design and print the surgical guides, and the cost burden of the owner increases. However, the surgical guides can be designed and printed in less than approximately half a day, and eventually produces more accurate surgical results. Furthermore, friction between the TPLO surgical guide and the drill and saw might produce microscopic debris. The material (Figure 4 MED-AMB 10, 3D systems Inc., Rock Hill, SC) used for the TPLO surgical guide was rigid and translucent and was biocompatible and autoclavable for medical use. The use of materials that meet the ISO 10993-5 and−10 standards for biocompatibility (cytotoxicity, sensitization, and irritation) and intraoperative lavage could mitigate the risk of side effects associated with microscopic debris. Further research is required on the unknown effects of the aforementioned material on postoperative wound healing.

The present study had some limitations. First, the study was not applied to clinical cases using cadaveric bones. This necessitates evaluating if similar results will be derived by applying them to clinical cases in future. Second, all osteotomies were performed by a single surgeon; although this approach has the advantage of not introducing surgeon-specific variations in the methodology, further evaluation of the influence of other surgeons is warranted. Additionally, it is necessary to determine if other commercially available jig systems, besides Slocum jig, will generate similar results. Fourth, the present study included a small number of cases. Further studies with dogs of various breeds are required to investigate the clinical relevance of TPLO surgical guides.

The technique presented in the present study may provide for an effective and easily applicable TPLO surgical guide using 3D printing technology. The use of the TPLO surgical guide was associated with more anatomically accurate osteotomy, compared to conventional TPLO, which may result in more precise alteration of the TPA. The use of the surgical guide may also reduce the operation time and possible complications from an inaccurate osteotomy. Further studies of larger cases in clinical practice are required to determine the clinical efficacy of this novel technique.

## Data Availability Statement

The original contributions presented in the study are included in the article/supplementary material, further inquiries can be directed to the corresponding author/s.

## Ethics Statement

The animal study was reviewed and approved by Institutional Animal Care and Use Committee of Konkuk University (Approval No. KU21039).

## Author Contributions

JayK: study conception and design, manuscript preparation, designing of the surgical guide, and data collection and interpretation. JaeeK: study conception and design. JaehK: study design and manuscript review. AS: designing and printing of the surgical guides. KE: support in all stages of manuscript preparation and review. All authors contributed to the article and approved the submitted version.

## Funding

This study was supported by Institute of Information & Communications Technology Planning & Evaluation (IITP) grant funded by the Republic of Korea Government (No. 2021-0-00839).

## Conflict of Interest

The authors declare that the research was conducted in the absence of any commercial or financial relationships that could be construed as a potential conflict of interest.

## Publisher's Note

All claims expressed in this article are solely those of the authors and do not necessarily represent those of their affiliated organizations, or those of the publisher, the editors and the reviewers. Any product that may be evaluated in this article, or claim that may be made by its manufacturer, is not guaranteed or endorsed by the publisher.
